# Effect of treatment with a full-occlusion biofeedback splint on sleep bruxism and TMD pain: a randomized controlled clinical trial

**DOI:** 10.1007/s00784-020-03270-z

**Published:** 2020-05-19

**Authors:** Alexander Bergmann, Daniel Edelhoff, Oliver Schubert, Kurt-Jürgen Erdelt, Jean-Marc Pho Duc

**Affiliations:** 1 Department of Prosthetic Dentistry, University Hospital, LMU Munich, Goethestraße 70, 80336 Munich, Germany; 2 Praxis Dr. Bergmann, Angerstr. 9, 83646 Bad Toelz, Germany

**Keywords:** Biofeedback/therapy, Occlusal splint, Sleep bruxism/therapy, Vibration, Temporomandibular disorders

## Abstract

**Objectives:**

The purpose of the present study was to analyze treatment outcome with a full-occlusion biofeedback (BFB) splint on sleep bruxism (SB) and TMD pain compared with treatment with an adjusted occlusal splint (AOS).

**Materials and methods:**

Forty-one patients were randomly allocated to a test (BFB) or a control (AOS) group and monitored over a 3-month period. Output variables were frequency and duration of bruxing events (bursts) and various pain symptoms.

**Results:**

The BFB group showed a statistically significant reduction in the frequency and duration of bursts and a statistically significant improvement in the patients’ global well-being and the facial muscle pain parameter. After the treatment was stopped, the BFB group showed a statistically significant reduction in the average and maximum duration but no statistically significant change in the frequency of bursts.

**Conclusions:**

The tested BFB splint is highly effective in reducing SB at the subconscious level, i.e., without waking the patient, and in achieving improvements in global pain perception. The results suggest that the BFB splint also provides a better treatment option for bruxism-related pain than an AOS. However, further research is needed, and specifically studies with a larger patient population displaying higher levels of pain at baseline.

**Clinical relevance:**

By reducing burst duration and therefore the pathological load on the masticatory apparatus, the BFB splint reduces TMD and bruxism-related symptoms and improves patients’ physical well-being. In the long term, this could prevent damage to the TMJ. This study confirms the effectiveness and safety of this splint.

**The universal trial number:**

U1111-1239-2450

**DRKS-ID registration:**

DRKS00018092

## Introduction

International bruxism experts have consensually defined bruxism as “a repetitive jaw muscle activity characterized by clenching or grinding of the teeth and/or by bracing or thrusting of the mandible” [1]. The American Academy of Sleep Medicine (AASM) has categorized sleep bruxism as “a sleep-related movement disorder” and defines it as “a stereotyped movement disorder characterized by grinding or clenching of the teeth during sleep” [2]. Bruxism has two distinct circadian manifestations: it can occur during sleep (indicated as sleep bruxism, SB) or during wakefulness (indicated as awake bruxism, AB) [1].

Several studies have demonstrated that bruxism might be a causal factor of various pain symptoms and functional limitations [3, 4] and a causal factor, or at least a risk factor, in TMD [5]. Still, the true relationship between these conditions and bruxism remains uncertain [4].

It is generally accepted that bruxism occurs at a parafunctional level and entails significant discomfort or damage to affected patients. A significant proportion of the population is affected [6]. The search for effective treatments is therefore an essential objective of scientific research.

Varying statistics have been presented regarding the prevalence of bruxism in the general population. This variation is largely due to the highly divergent experimental setups of investigations engaged with the subject. It has been reported that between 8% and 8.6% of adults exhibit “frequent” bruxism and 31.4% exhibit “some” bruxism, irrespective of its frequency [4, 7–9].

Biofeedback (BFB) has been described as a technique that provides individuals with information about the condition or activities of their bodies, with the intention of promoting changes in behavior that result in improved health or performance [10, 11]. The objective is to make the subjects aware of harmful processes, enabling a countervailing response to avoid the undesired effect. The method is mostly used in the waking state. As the neurological mechanisms related to AB differ from those of SB [7], unconscious grinding and clenching while sleeping cannot be actively inhibited by individual willpower. It may, however, be possible for subjects to learn to react to biofeedback even while sleeping [12].

Numerous studies have been conducted on bruxism-related symptoms, its management, and the effect of biofeedback on SB. Reviews frequently mention study limitations and, hence, reasons to call for further research, including small sample sizes, a lack of randomization, inadequacies in blinding methodologies, concealment of allocation, handling of withdrawals and losses, selective or incomplete reporting, short test periods, the absence of a control group, a lack of adequate baseline data, and shortcomings in outcome reporting [13–17].

There may be many reasons why studies would report different results, some of them related to study design. However, even within a given study, different results have been reported for different forms of biofeedback [13]. This would suggest that we cannot assume that all forms of biofeedback will have the same effect. The many different approaches make it difficult to draw definitive conclusions [18]. Whatever new type of biofeedback treatment becomes available should therefore invariably be the subject of critical evaluation.

Most accessible studies dealing with the effect of biofeedback on bruxism have measured muscular (m. temporalis and/or m. masseter) activity using external electrodes linked to an electromyograph, with the biofeedback stimulus being delivered externally, e.g., using speakers or supracutaneous electrical impulses [17].

Through the incorporation of a pressure sensor and microcontroller, the tested splint can be used to measure bruxing events during sleep. A device using a similar activation/deactivation system in an occlusal splint was assessed by other researchers and validated against electromyograph (EMG) measurements. It was established that the operating principle of the tested splint is valid [19].

Unlike previous treatment modes, the present approach entails a dental splint incorporating a vibratory stimulus with an additional auditory alarm. This is supposed to have several relevant implications:

- The occlusal splint by itself may influence the treatment.
- The biofeedback is triggered directly by the occlusal force applied, not indirectly by the measurement of muscular activity.
- The delivery of the biofeedback stimuli is intrabuccal, i.e., delivered at the time and location of the targeted activity.

A pilot study by Gu et al. [20] tested a dental splint with extrabuccal biofeedback and reported a significant reduction in the frequency and duration of bruxing events, but did not assess the effect on the level of pain. Hara et al. [14] tested an occlusal splint with intrabuccal biofeedback and reported a reduction in SB frequency, but did not examine the duration of bruxing events. The authors reported that there was no significant impact on the level of pain.

The present study attempts to evaluate a recently introduced new biofeedback treatment using a larger sample, comprehensive data collection of a larger number of outcome variables, and a longer observation period.

Despite the emergence of new therapies, the most prevalent treatment for bruxism remains the adjusted acrylic occlusal splint (AOS). This applies to Germany [21] as well as to other countries [16, 22, 23]. The literature, however, shows ambivalent results concerning its effectiveness [24].

In this context, the objective of this study was to test the following hypotheses:

1. Treatment with the biofeedback splint reduces the number and duration of bruxing events.
2. Treatment with the biofeedback splint leads to a significantly better improvement in symptoms compared with the control group (AOS group).
3. The post-treatment impact of the biofeedback is higher than in the AOS group.

An additional objective was to monitor for any adverse effects, particularly any that exceeded those associated with the AOS.

## Materials and methods

### Subjects

Patients older than 18 years displaying TMD and signs of bruxism who sought treatment at the Department of Prosthetic Dentistry, University Hospital, Ludwig Maximilian University of Munich, Germany were recruited. Recruitment and follow-up assessment took place from February 2016 to July 2018.

### Inclusion criteria

1. Physical signs of bruxism visible on the dentition (grinding facets, abnormal tooth wear, or wedge-shaped lesions)
2. Self-reported pain in the masticatory muscles or the temporomandibular joint (TMJ)
3. Willingness of the patient to participate in the study and a commitment to adhere to the pre-set timetable
4. Measured SB activity

### Exclusion criteria

#### General medical criteria

1. Acute pain caused by other components of the masticatory system (e.g., caries, root inflammation)
2. Prior or planned TMJ or dysgnathia surgery
3. Jaw fractures
4. Otorhinolaryngologic diseases (except tinnitus)
5. Systemic basic illness with rheumatic origin (e.g., arthritis, arthrosis, gout, and psoriasis)
6. Psychosomatic or psychiatric diseases
7. Implanted electronic devices
8. Arrhythmia and other (prior or present) cardiac problems
9. Epilepsy
10. Cerebrovascular and brain diseases
11. Pregnancy including breastfeeding period
12. Drug or alcohol abuse, analgesic, or sedative therapy, use of medication affecting the central nervous system (e.g., antidepressants, anxiolytics, and anticonvulsants)
13. Physical or mental disability

#### Criteria specific to the study

(14) Maxillary hyperesthesia or allergy to materials used
(15) Missing support zones in the posterior region
(16) Anatomical topography that made a full-coverage maxillary splint impossible to use
(17) Anatomical topography that did not tolerate the increased vertical dimension of occlusion
(18) Past history of received biofeedback therapy
(19) Insufficient recorded bruxing activity in the baseline phase

The above inclusion and exclusion criteria were defined under medically and scientifically meaningful scrutiny and differ from those stated by the device’s manufacturer. The manufacturers’ criteria were handed to each study participant with the device’s manual. The study environment enabled a higher degree of medical supervision than would normally be available to patients. Thus, the sample for the study could be drawn from a slightly broader population. Tooth or jaw misalignments, which would have made it impossible to produce a functional splint, did not occur.

**Fig. 1:**
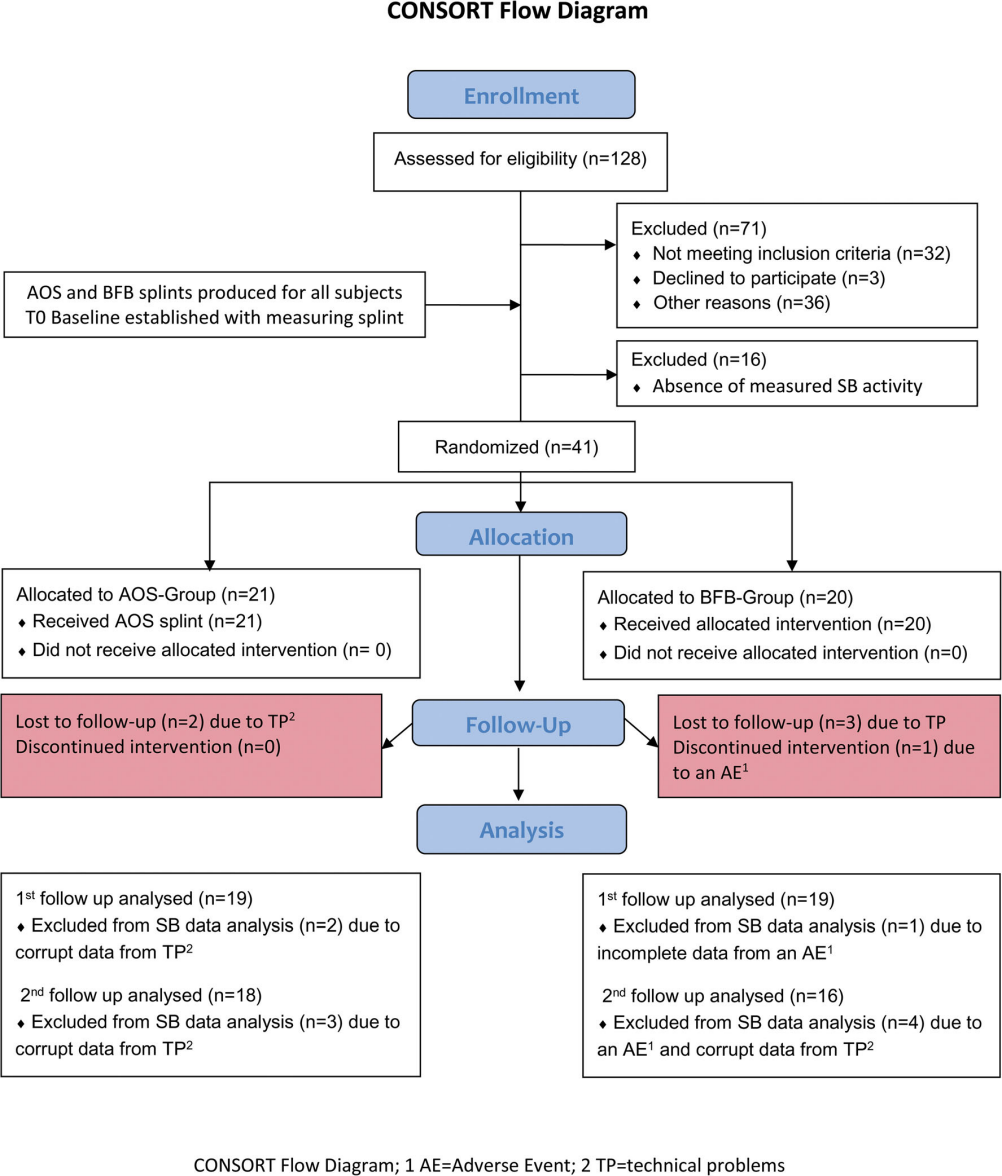
CONSORT flowchart (Word)

Following application of the exclusion criteria (see Fig. 1), a sample of 41 patients formed the trial population. Insufficient recorded SB activity, medication, and the absence of compatible computer hardware were the main reasons for exclusion. All participants were instructed in the use of the splints and asked to report any use of alcohol or medicines on their daily control sheets, as these can dampen the response to biofeedback stimuli.

Three subjects from the AOS group and three subjects from the BFB group did not complete the test as their biofeedback splints (in the case of the AOS group subjects, for measurement purposes) developed technical problems. Their data could be used up to and including phase E2 (see Table 1).

### Randomization and blinding

Subjects meeting inclusion criteria were randomly allocated by selection of sealed opaque envelopes to either of two groups: a control group using a conventional occlusal splint (AOS group) and a test group using the biofeedback splint (BFB group). The recruiter had no information about the allocation pattern. As there were obvious physical differences between the control and test splints and it was unavoidable that the patient could easily identify whether the biofeedback splint was in applied (i.e., biofeedback mode), patient-side blinding was not possible.

Because of resource limitations, one member of the university staff (a qualified dentist) conducted the study and data appraisal. There was no therapist-side or analyst-side blinding.

### Description of the devices

Maxillary splints were used for both the AOS and BFB groups. All devices were made by the same technician in a dental laboratory (Dentaltechnik Michael Seitz, Munich, Germany). As all splints were manufactured prior to randomization, a conventional and a biofeedback splint were produced for each subject; irrespective of the group, the subjects were finally allocated to. BFB splints (with the biofeedback switched off) were needed for the AOS group as well since they would be worn as a measuring tool (see the “Study design” section below). The Michigan splints produced for the BFB group were discarded unused. By manufacturing both splints for all subjects prior to randomization, we intended to remove any possible manufacturing bias and to ensure adherence to the predetermined timetable.

**Fig. 2:**
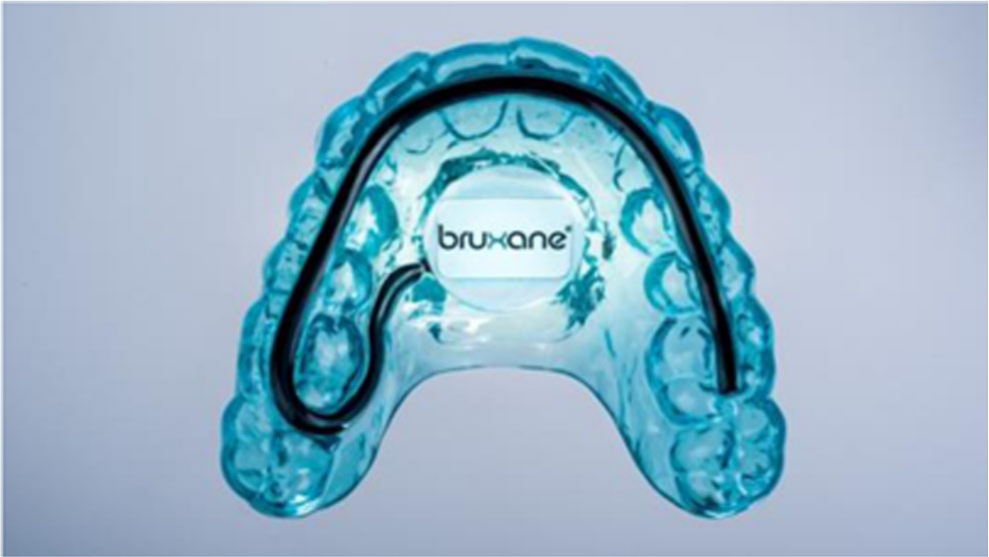
Biofeedback splint (BFB)

The BFB splint used in the study differs from the version the manufacturer sells today. The splint in this study had a silicone contact tube as sensor (Fig. 2). The commercial version uses a sensor that is approximately 0.75 mm thinner. From a technical point of view, the manufacturer has reduced the VDO extension of the BFB splint.

All AOS and BFB group splints had a flat plane with homogeneous occlusal contacts in centric relation, with anterior guidance for excursive moments.

Analog maxillary and mandibular impressions, interocclusal bite registration, and facial arch transfer/facebow records (Arcus; KaVo Dental, Biberach, Germany) were obtained under constant conditions by the same team of skilled professionals. Plaster casts were manufactured, analyzed, and mounted in a semiadjustable articulator (KaVo EWL; KaVo Dental).

**Fig. 3:**
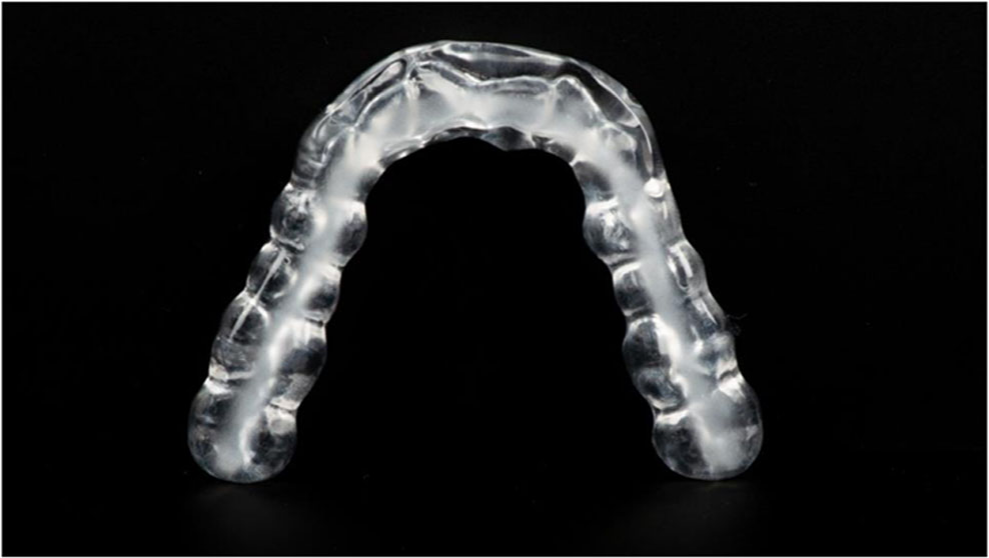
Adjustable occlusal splint (AOS)

The splint used by the AOS group was made of clear autopolymerizing dental acrylic resin (Orthocryl; Dentaurum, Ispringen, Germany) (Fig. 3).

The bruXane splint (bruXane, Marburg, Germany) used by the BFB group (Fig. 2) was made of two soft thermoformed full-coverage maxillary dental plates (bruXflex; Erkodent, Pfalzgrafenweiler, Germany). A pressure-sensitive sensor was integrated along the entire occlusal surface, with electronic components housed in the palatal area, including a rechargeable battery, a vibrating motor, and a microcontroller.

The microcontroller continuously monitored the resistance level in the sensor. Occlusal pressure on the sensor reduced the electrical resistance. When the resistance fell below a predetermined threshold level, the microcontroller classified this as the start of a bruxing event (burst) and simultaneously switched on the vibrating motor. Releasing the occlusal pressure reversed the process, which the microcontroller recognized as the end of the burst and stopped the vibrating. The minimum measurable burst duration was 100 ms; longer bursts were measured in 100-ms increments.

The threshold for triggering the microcontroller was calibrated and documented during production and was equivalent to 16.1 ± 5.1 kg (mean ± SD) across all subjects. At this level, normal activities, such as myoclonus, swallowing, or coughing, would not trigger a response.

Both splints were examined before being handed over to the patients as well as at each follow-up. The AOS splints were contoured before being issued to the patient, initially and at each follow-up if necessary. Adjustments to the BFB splints were not permitted, so as to avoid the risk of moisture entering the electronics. If any unevenness of the occlusal surface was discovered when fitting a splint for a patient, a new bite registration was taken and sent to the dental laboratory along with the splint for adjustment (if possible) or remake. No adjustments of the surface of the BFB splint occurred after data collection had started.

Other researchers have described a device using a similar activation/deactivation system and validated this approach against electromyograph (EMG) evidence [25].

### Study design

The baseline situation was established as follows:

#### Bruxing (quantitative) data

Both groups wore the biofeedback splint in recording-only mode for approximately 2 weeks. A pilot trial showed that the first few nights would display abnormally low bruxing activity before a more regular pattern was re-established. As already stated by Klasser et al., insertion of an unfamiliar object in the mouth can interrupt the regular bruxing pattern [23]. Therefore, the first four nights´ baseline data were excluded from the analysis.

#### Symptoms (qualitative) data

The assessment consisted of a questionnaire survey and a clinical examination according to the research diagnostic criteria for temporomandibular disorders (RDC/TMD) Axis I and II. The treating dentist was trained in TMD diagnosis in accordance with the German version of the RDC/TMD manual [26, 27]. To evaluate a treatment effect, 4.5 months can be considered appropriate [28].

Table 1 summarizes the treatment and measurement phases.

**Table 1:**
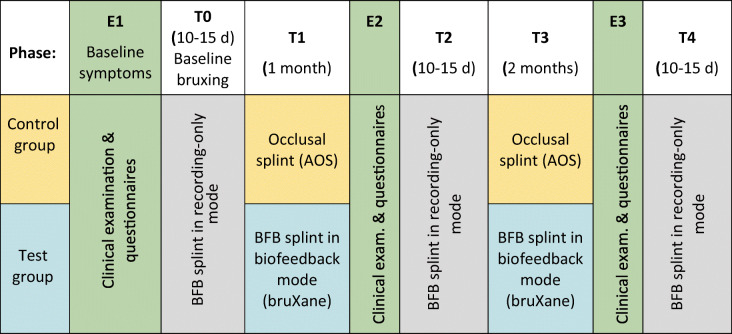
Timetable for the treatment and measurement phases

### Data collection

#### Bruxing (quantitative) data

Pseudonymized data collection was continual, not just sampled, i.e., during each phase (measurement phases for the AOS group and measuring and treatment phases for the BFB group), every burst during every sleep period was recorded.

The data stored in the microcontroller was periodically transferred by the subject to a computer as a .csv file. This file was sent to the Ludwig Maximilian University of Munich and analyzed using proprietary software (bruXane, Marburg, Germany), which calculated the following *outcome variables* per subject and sleep period (i.e., per night) from the raw burst data:

##### Total duration per hour

The sum of the durations of each burst divided by the number of hours of the respective sleep period, reported in seconds. This is representative of bruxing activity.

##### Bursts per hour

The number of bursts divided by the number of hours of the respective sleep period.

##### Average duration

The sum of the durations of each burst divided by the number of bursts in the respective sleep period, reported in milliseconds.

##### Maximum duration

The duration of the longest individual burst in the respective sleep period, reported in milliseconds.

**Fig. 4:**
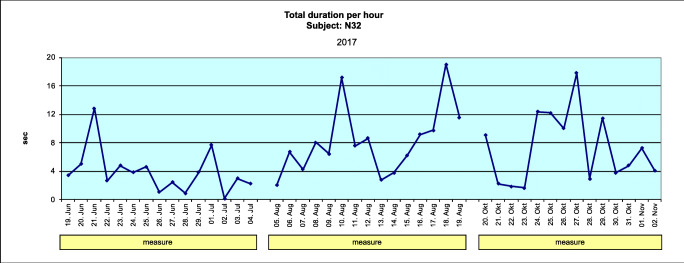
Mean total duration per hour for the AOS group (adjustable occlusal splint), obtained during one night (average subject) (Excel)

**Fig. 5:**
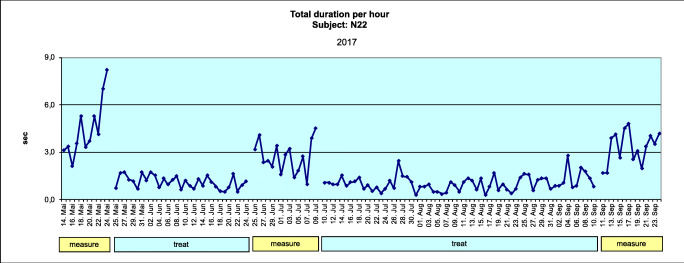
Mean total duration per hour for the BFB group (biofeedback splint), obtained during one night (average subject) (Excel)

Figures 4 and 5 show data for the total duration per hour (TDPH) for a typical subject (where the subject mean change is equivalent to the group mean change) in the AOS and BFB groups, respectively.

#### Symptom (qualitative) data

Using questionnaires, the subjects evaluated various specific pain and functional symptoms on an NRS scale of 0 to 10 at three discrete points in time: immediately before testing, after 1 month of treatment, and after the second treatment phase of another 2 months. Additionally, subjects reported on their global pain perception before and after testing.

#### Clinical examination

Clinical examinations were undertaken at the time of the questionnaire surveys. RDC criteria and recommendations were met and the examination was conducted by the same skilled professional [26, 27].

### Statistical analysis

All data were analyzed with SPSS Statistics 25 (SPSS, Stanford, CA, USA). The level of significance was set at *p* < 0.05. The Kolmogorov-Smirnov test was used to test the data for normal distribution. Descriptive statistics (means and SD) were calculated. If the assumption of normality was true, the Student *t* test was performed. If it was not, the Wilcoxon signed-rank and Mann-Whitney *U* tests were performed.

Non-parametric tests were used for the questionnaire data. Between groups, the analysis was based on the pre/post differences within each group. The primary concern was to analyze each subsequent phase against the respective baseline (phase T0 or E1, as appropriate).

### IMMPACT recommendations

Cognizant of the recommendations of the Initiative on Methods, Measurement, and Pain Assessment in Clinical Trials (IMMPACT) [29], we assessed whether changes in the patients’ reported symptoms were clinically meaningful, defined as a change of greater than 30% in the pre/post mean values.

## Results

### Demographic data (Table 2)

**Table 2:** Mean age and gender distribution in the AOS group (adjustable occlusal splint) and the BFB group (biofeedback splint)

	Control	Test	*p*
Mean age	41.3 ± 14.2	37.6 ± 11	0.701 (Fisher exact test)
Gender			
n	20	19	
Female	11 (55%)	10 (52.6%)	
Male	9 (45%)	9 (47.4%)	

### Baseline phases (T0 and E1)

There was no statistically significant difference between the AOS and BFB groups at baseline, whether bruxing (quantitative) data or symptom (qualitative) data. On a scale of 0 to 10, the mean baseline value for all symptom/limitation outcomes for both groups was 2.92 ± 1.22.

#### RDC/TMD Axes I and II

Research diagnostic criteria for temporomandibular disorders (RDC/TMD) were applied to baseline data and used to detect and classify the diseases of all test subjects. No significant differences were found between the two groups regarding Axes I and II.

### Overview of the measurement and treatment phases (T0 to T4)

**Fig. 6:**
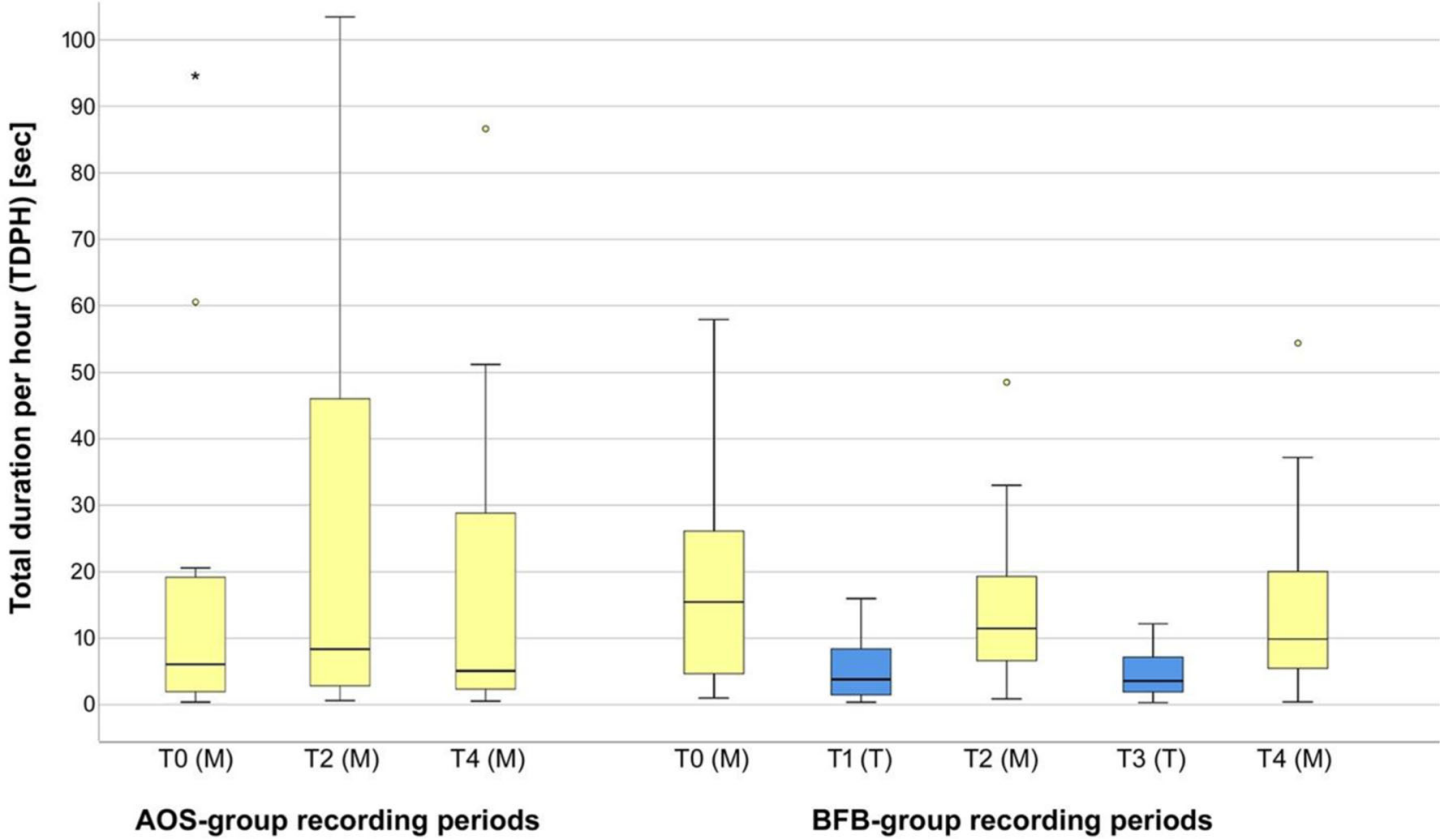
Box plots of the total duration per hour for all phases of the AOS group (adjustable occlusal splint, left) and the BFB group (biofeedback splint, right) (SPSS)

The box plots in Fig. 6 show the nightly total bruxing duration per hour. The plot on the left shows the total duration per hour for all three AOS group measurement phases at baseline (T0), the first follow-up (T2), and the second follow-up (T4) (see also Table 1). Comparable measurements during the treatment phases for the AOS group were not possible due to the study design. There was considerable variance in each of the phases and there were similar mean values across all the phases in the AOS group.

The plot on the right also shows all three measurement phases (yellow bars) for the BFB group and the two intermediate treatment phases (blue bars, T1 and T3) with significantly lower mean values and significantly less variance during the treatment phases with biofeedback.

### Treatment phases (T1 and T3)

#### Examination of hypothesis #1

##### Treatment with the biofeedback splint reduces the number of bruxing events and their duration

For each outcome variable, normality of distribution at baseline (T0) and each of the two treatment phases (T1 and T3) was tested. For phase T3, data were available for only 17 subjects. These data were compared with the T0 data for the same subjects. So T0 (*n* = 19) was compared with T2 (*n* = 19) and T0 (*n* = 17) with T3 (*n* = 17). The T0 data were always the same, but in the latter case, they applied to a subset of the subjects. The results showed normal distribution for total duration per hour, bursts per hour (BPF), and average duration (AD). Maximum duration (MD) showed no normal distribution. A Student *t* test was therefore performed for the variable’s total duration per hour, bursts per hour, and average duration, while the Wilcoxon signed-rank test for paired samples was performed for maximum duration.

The results of the statistical tests are given in Table 3.

**Table 3:** Statistical tests for bruxing activity within the BFB group (biofeedback splint)

Phase	T0 (*n* = 19) measurement	T1 (*n* = 19) treatment	*p* value	T0 (*n* = 17) measurement	T3 (*n* = 17) treatment	*p* value
TDPH (s)^a^	19.1 ± 16.1	5.2 ± 4.5	0.001	19.6 ± 16.6	4.4 ± 3.5	0.001
BPH (units)^a^	21.3 ± 14.5	12.9 ± 10.7	0.002	21.3 ± 14.1	12.9 ± 10.0	0.010
AD (ms)^a^	868.4 ± 315.2	374.8 ± 110.9	< 0.001	879.6 ± 329.8	328.9 ± 94.4	< 0.001
MD (ms)^b^	11,671 ± 12,839	3526 ± 4407	< 0.001	12,418 ± 13,405	2366 ± 2233	< 0.001

^a^Student’s paired *t* test

^b^Wilcoxon signed-rank test

The table shows the means and standard deviations for each parameter/phase combination. Statistically significant reductions (see the *p* values) were evident for all outcome variables in the first treatment phase (T1) within the BFB group. These were maintained or slightly improved in the second treatment phase (T3).

### Evaluation phases (E2 and E3)

#### Examination of hypothesis #2

##### Treatment with the biofeedback splint leads to a significant improvement in patient symptoms compared with the control group

Statistical testing for symptoms and functional limitations yielded two outcomes for which the BFB group showed statistically significant improvements over the AOS group by the end of testing (E3): general pain perception (*p* = 0.017) and pain in the facial muscles (*p* = 0.038). There were no outcomes for which the AOS group performed statistically better than the BFB group.

Following IMMPACT recommendations [29], Table 4 summarizes the within-group changes in symptom severity for which either group showed a clinically meaningful (30%) improvement (below the line) or deterioration (above the line). Negative percentages indicate a reduction in symptom severity (i.e., an increase in patient well-being) and vice versa.

**Table 4:**
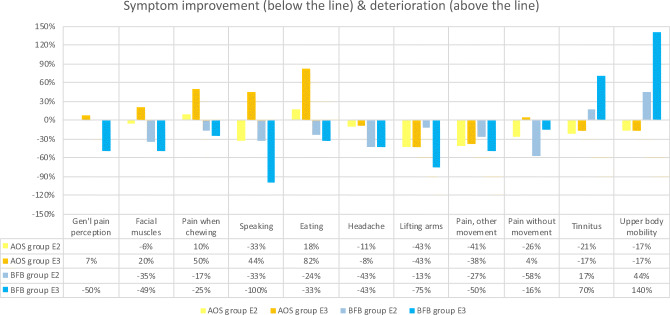
Changes in symptom severity for the AOS group (adjustable occlusal splint) and the BFB group (biofeedback splint)

For the symptoms most strongly associated with bruxing (facial muscles, chewing, speaking, and eating), the biofeedback splint produced meaningfully better results than the AOS splint.

### Measurement phases (T2 and T4)

#### Examination of hypothesis #3

##### The post-treatment impact of the biofeedback is larger compared with the control group

Comparing the BFB group and the AOS group produced the following results (green *p* values are statistically significant):

Table 5 shows changes in group means and standard deviations between the phases. Increases in means from the earlier to the later phase are shown in red. The *p*xvalues are the result of statistical tests on differential changes between the groups. Total bruxing activity (total duration per hour) showed a statistically significant improvement in the BFB group compared with the AOS group in phase T2.

**Table 5:**
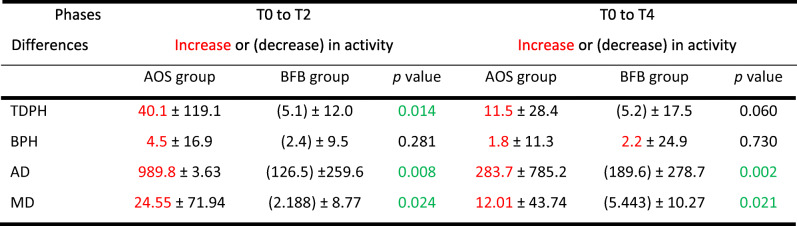
Statistical testing for bruxing activity for the AOS group (adjustable occlusal splint) and the BFB group (biofeedback splint)

The BFB group showed a statistically significant improvement in duration of bursts (both average and maximum) compared with the AOS group in both post-baseline measurement phases. No statistically significant difference was found between the groups in terms of the frequency of bursts (bursts per hour).

## Discussion

### Treatment phases (T1 and T3)

#### Hypothesis #1

##### Treatment with the biofeedback splint reduces the number of bruxing events and their duration

On the basis of the data, the hypothesis is accepted.

**Fig. 7:**
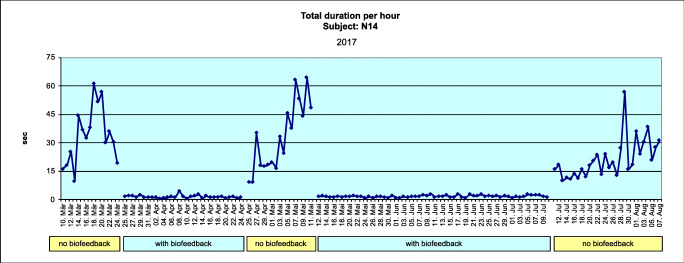
Total duration per hour, BFB group (biofeedback splint) treatment, and recording periods (Excel)

There was a statistically significant reduction in total bruxing activity (total duration per hour) during the treatment phases with the biofeedback splint compared with baseline (T0). The second treatment phase (T3) showed few changes from the first treatment phase (T1), which indicates that the effect of the biofeedback occurs early and is then sustained. An extreme illustration of this phenomenon is subject N14 (see Fig. 7).

Of the two components of total duration per hour, both the frequency (bursts per hour) and average duration (AD) of bursts significantly declined.

This study did not involve the subjects being woken up by the biofeedback stimuli. By comparing patients and bed partners´ reports of incidents of being awakened through the biofeedback with the number of recorded bruxing events (and, hence, the presence of biofeedback stimuli), it was found that the incidence of subjects awaking from the biofeedback was negligible.

A clinically relevant finding of this study is that the body is effective in responding to biofeedback at the subconscious level. It should be noted that the biofeedback was delivered to the patient directly at the point of the bruxing activity in the form of two stimuli (vibration and sound), i.e., two senses are addressed. This is different from the biofeedback applied in most other studies and may indicate that the type of biofeedback system in use may be a relevant factor, i.e., that not all biofeedback methods have the same effect. Further studies must be conducted to verify this conclusion.

While the tested biofeedback splint does not measure the force applied during bruxing, it is likely that longer bursts produce greater forces (e.g., when comparing the highest recorded patient mean of 55,445 ms maximum duration with the lowest recorded patient mean of 1643 ms). Maximum burst duration could therefore serve as a proxy for measuring the stress on the stomatognathic system. As shown in Table 3, there was a significant reduction in the maximum duration of bursts during the treatment phases, which could significantly reduce the total level of stress on the stomatognathic system.

### Evaluation phases (E2 and E3)

#### Hypothesis #2

##### Treatment with the biofeedback splint leads to a significant improvement in patient symptoms compared with the control group

The hypothesis is accepted in respect of patients’ global pain perception and pain in the facial muscles. The AOS showed no statistically significant improvement over the biofeedback splint regarding any symptoms. No statistically significant conclusions were drawn in respect of the other parameters.

The literature suggests that the AOS leads to an improvement in symptoms in the case of TMD and bruxism [16, 23, 30]. Such a conclusion was not confirmed by the results of the present study.

A limitation of the study was that the number of subjects reporting specific symptoms was very low in some cases, so conclusions must be drawn cautiously. For symptom evaluation, unlike the objective measurement of bruxing activity, the lack of blinding in this study might have biased the outcome values.

Future studies should focus on subjects displaying more intense symptoms/functional limitations. The data reveal that there is a tendency for symptom reduction to be greater when baseline values are higher and that the reduction is greater, the longer the therapy phase lasts. Given the inherently subjective nature of these qualitative tests, the differences within and between the groups may be more significant when the baseline level is higher. Furthermore, the disparate performance of the two treatment methods on different outcome variables highlights the importance of including a wide range of pain/functional limitation variables in any research.

### Measurement phases (T2 and T4)

#### Hypothesis #3

##### The post-treatment impact of the biofeedback is higher than compared with the control group

On the basis of the data, the hypothesis is accepted.

The reduction in the test group compared with the control group was statistically significant for the duration of bursts (both average and maximum). Whereas the test group bursts per hour were reduced in phase T2, they increased in phase T4. These differences were not, however, statistically significant.

The question arises to what extent the measurement method (use of the biofeedback splint in recording-only mode) could have influenced the results, particularly regarding the increased activity in the control group. This outlines a practical limitation of the present study design. Nevertheless, the post-treatment measurement method was identical between the groups, and we would expect any measurement bias to apply equally to the test group and the control group, also resulting in an increase in post-treatment bruxing activity in the test group. The fact that the test group showed a reduction regarding most variables suggests that there was a long-lasting effect even after the biofeedback treatment was discontinued.

Another possible factor was the differential vertical dimension of occlusion (VDO) between the two types of splint. For technical reasons, the VDO for the biofeedback splint—unlike for the AOS—was at least 6–10 mm. A reduced VDO is associated with greater wear comfort and reduced muscular activity [31]. This effect was not tested for, but it could have influenced the results.

The results should be viewed within the context that there was an increase in VDO in the AOS group when switching from the AOS splint to the recording splint in the follow-up phases (T2 and T4). This may have influenced the results. Further studies should rely on EMG measurements to avoid this problem.

The results show that there was a significant reduction in all bruxing outcomes during biofeedback treatment. The post-treatment reduction primarily affected the duration of bruxing events. In the present study, the treatment phases were limited to 1 month and 2 months, respectively. Because biofeedback as a therapy is a “process of training” [11], where patients have to “unlearn their behavior” [17], a greater post-treatment impact may be expected after a longer treatment phase, possibly outperforming any effect achievable using a “passive” splint. Further studies should explore the long-term effects of this combination of splint and biofeedback therapy. As part of such a study, it would be of interest whether there is a correlation between the length of the bruxing history and the length of the treatment phase required to obtain lasting post-treatment effects.

In the present study, the post-treatment measurement phase was limited to 2 weeks immediately following the treatment phase. Further studies are required to explore the post-treatment effect regarding long-term effects.

### Changes in mandibular position

If the splint therapy caused relaxation and the patients were initially in a compressive state of the TMJ (centric relation that positions the mandible further cranially and dorsally), changes in the mandibular position could have occurred.

The position of the temporomandibular joints was not examined in the present study, and no imaging techniques were used. Therefore, no statement can be made about possible changes in compression. In the BFB group in particular, no adjustment to the splint was undertaken; hence, the position was not changed. Further studies are necessary to investigate this issue.

### Vertical dimension of occlusion

Another issue is whether and how the greater vertical dimension of occlusion (VDO) could have affected the recorded bruxing data. The results show that biofeedback therapy was effective and that both frequency and duration of bruxing events were reduced. It is possible that a greater VDO that interferes with the physiologic rest position could cause greater muscular activity. If that were the case, one might expect to record higher bruxing activity. The reduction in bruxing activity seen in the study probably understates the biofeedback effect.

No complications arose and no problems were reported by any patient due to the increased VDO. A few subjects reported increased snoring and sleeping with their mouth open.

### Adverse events

One subject (N6) in the BFB group reported diffuse neuralgic pain in the region of the right maxillary nerve after three nights of active biofeedback treatment. The subject wished to be excused from further participation in the study. This case was registered as the only adverse event (AE). There were no cases that could be classified either as serious adverse events (SAEs) or as suspected unexpected serious adverse reactions (SUSARs).

### Dropouts

As the biofeedback splint was partly used purely for measurement purposes, this led to excessive material wear and ingress of moisture during the measurement phase without biofeedback in six cases. In these cases, subsequent data were excluded and considered “technical problems”. This operating mode of the BFB splint is not used commercially and was developed exclusively for use in studies.

### Discussion summary

- The applied biofeedback therapy led to a significant reduction in bruxing activity in terms both of frequency and of duration.
- Treatment with the biofeedback splint led to a statistically significant improvement in patients’ pain perception, both globally and specifically in relation to facial muscles, also in comparison with the control group. With respect to other symptoms, the conclusions that could be drawn were limited by the small number of the data sets.
- The post-treatment impact of the biofeedback phase was a statistically significant reduction in the duration of bruxing events. Long-term effects should be investigated.

## Conclusion

### Bruxing activity

The tested biofeedback splint achieved a statistically significant reduction in bruxing activity during the treatment phase, in terms of the number of events and, especially, their duration; there were no adverse effects. While this treatment does not address the underlying cause of the patient’s bruxism, this appears to be an effective and safe tool within bruxism treatment. The measurement feature of the biofeedback splint may help identify the causes of the individual patient’s bruxism and the extent to which the reported symptoms are due to bruxing.

It was also seen that this biofeedback system seems to function effectively at the subconscious level (i.e., it does not wake the patient). Furthermore, the results suggest that the biofeedback splint reduces the bruxism-related level of stress on the stomatognathic system, potentially preventing other disorders that result from the application of bruxism-related forces.

### Symptoms and functional limitations

The biofeedback splint provides a statistically significant improvement in patients’ general well-being compared with the conventional AOS.

Treatment with the biofeedback splint led to statistically significant reduced pain in the facial muscles compared with the control group. The control group showed no statistically significant improvement over the test group regarding any of the variables tested.

Due to the limited number of patients who reported individual symptoms, further research is needed to validate these initial findings.

### Post-treatment effects

There is evidence that treatment with the biofeedback splint achieved a statistically significant reduction in the average and maximum duration of bursts even after treatment was stopped. Such reduction was not observed for the number of bursts. It can be hypothesized that few but extended bursts, which imply the application of greater force, have a more detrimental effect on the stomatognathic system than more but shorter bursts. If this is the case, the observed reduction in the duration of bursts is the more significant factor in terms of patient well-being.

### Conclusion summary

The biofeedback splint seems safe for therapeutic use and offers significant benefits to patients. Both splints examined protect the teeth. By reducing burst duration and, hence, the pathological load on the masticatory system, the biofeedback splint offers additional positive treatment effects in terms of prophylaxis and in reducing existing bruxism-related symptoms.
